# Supplementary material for: Ferrous Sulfate-Mediated Control of Phytophthora capsici Pathogenesis and Its Impact on Pepper Plant
Source: Plants (Basel). 2023 Dec 15;12(24):4168. doi: 10.3390/plants12244168 (PMC10748253; doi:10.3390/plants12244168)
Supplement: Supplementary file 1 [file plants-12-04168-s001.zip › plants-2740966-supplementary.pdf]

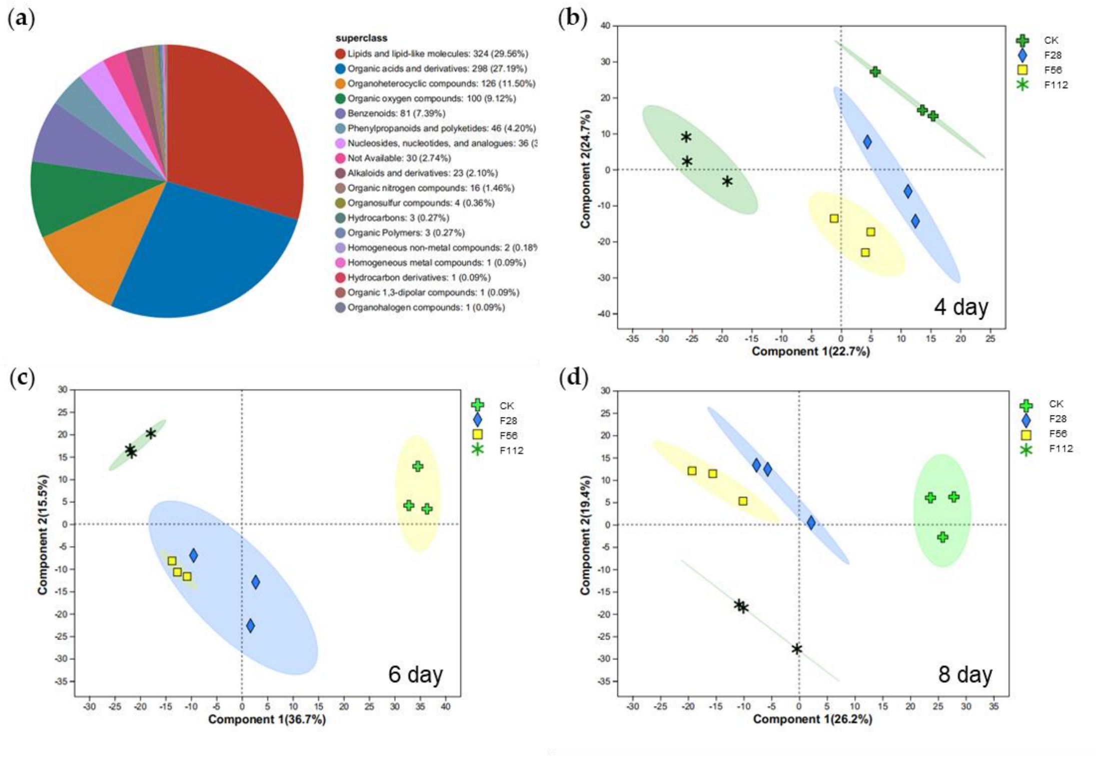

Supplementary Figure S1. Metabolite Classification, Counts, and Percentage Representation. (a) Classification of metabolites and the corresponding number and percentage distribution. (b-d) Partial Least Squares Discriminant Analysis (PLS-DA) at different time points (4 days, 6 days, 8 days) for FeSO4 treatments at concentrations of 0, 28, 56, and 112 mg/L.

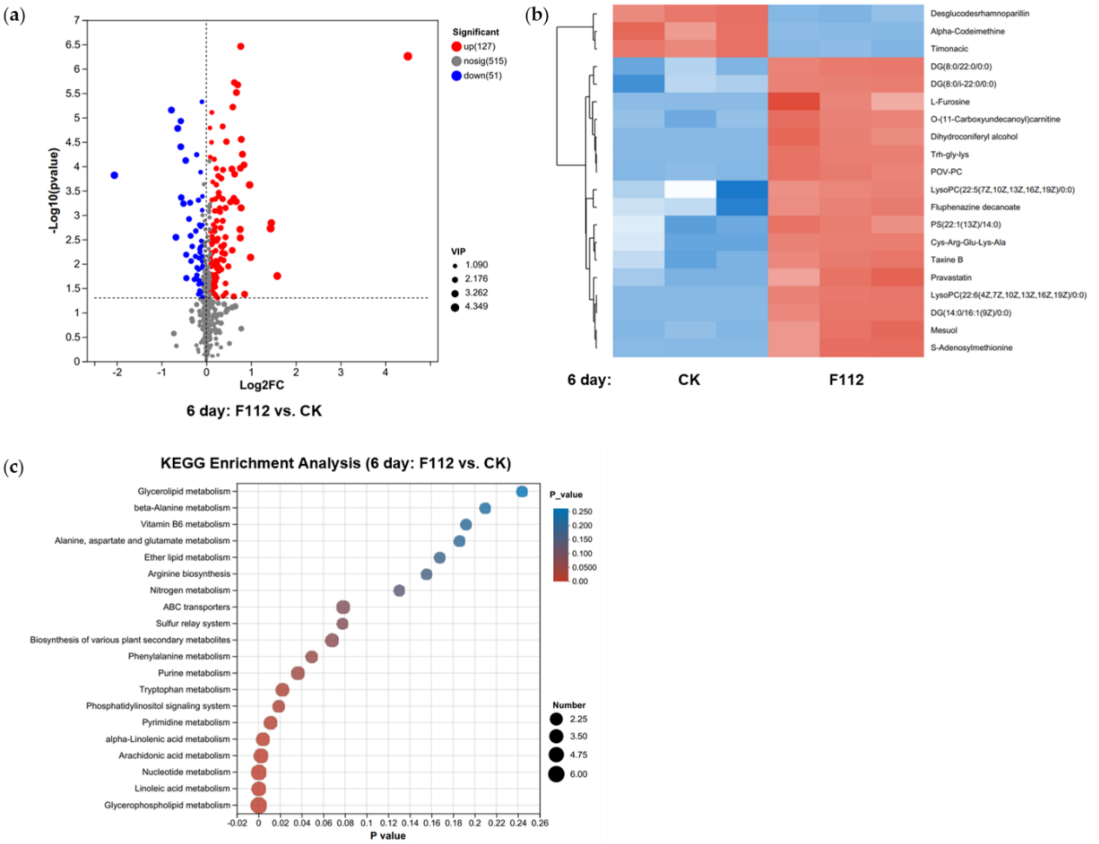

Supplementary Figure S2. Clustering Analysis of Differentially Expressed Metabolites between the F112 and CK Groups at 6 Days. (a) Volcano plots depicting differentially expressed metabolites between the F112 and CK groups at 6 days. The horizontal axis represents the log2 fold change (log2FC) in metabolite expression between the two groups, while the vertical axis represents the statistical significance of differences in metabolite expression (-log10(p_value)). Larger points indicate higher Variable Importance in Projection (VIP) values. Points on the left side represent downregulated metabolites, while points on the right side represent upregulated metabolites. The further to the left or right, and higher up on the plot, the more significant the expression difference. (b) Hierarchical clustering dendrograms illustrating the clustering of differentially expressed metabolites between the F112 and CK groups at 6 days. Closer branches indicate similar ex-pression patterns for all metabolites within the samples. Each column represents a sample, with sample names shown below. Each row represents a metabolite, with color indicating the relative expression level in the respective group, and the color gradient is depicted in the gradient color bar. (c) Pathway enrichment plots for the F112 and CK groups at 6 days. The horizontal axis represents the significance p-value of the enrichment, with lower p-values indicating greater statistical significance ($p < 0.05$ indicates significant enrichment). The vertical axis represents KEGG pathways. The size of bubbles in the plot represents the degree of enrichment of compounds in that pathway.

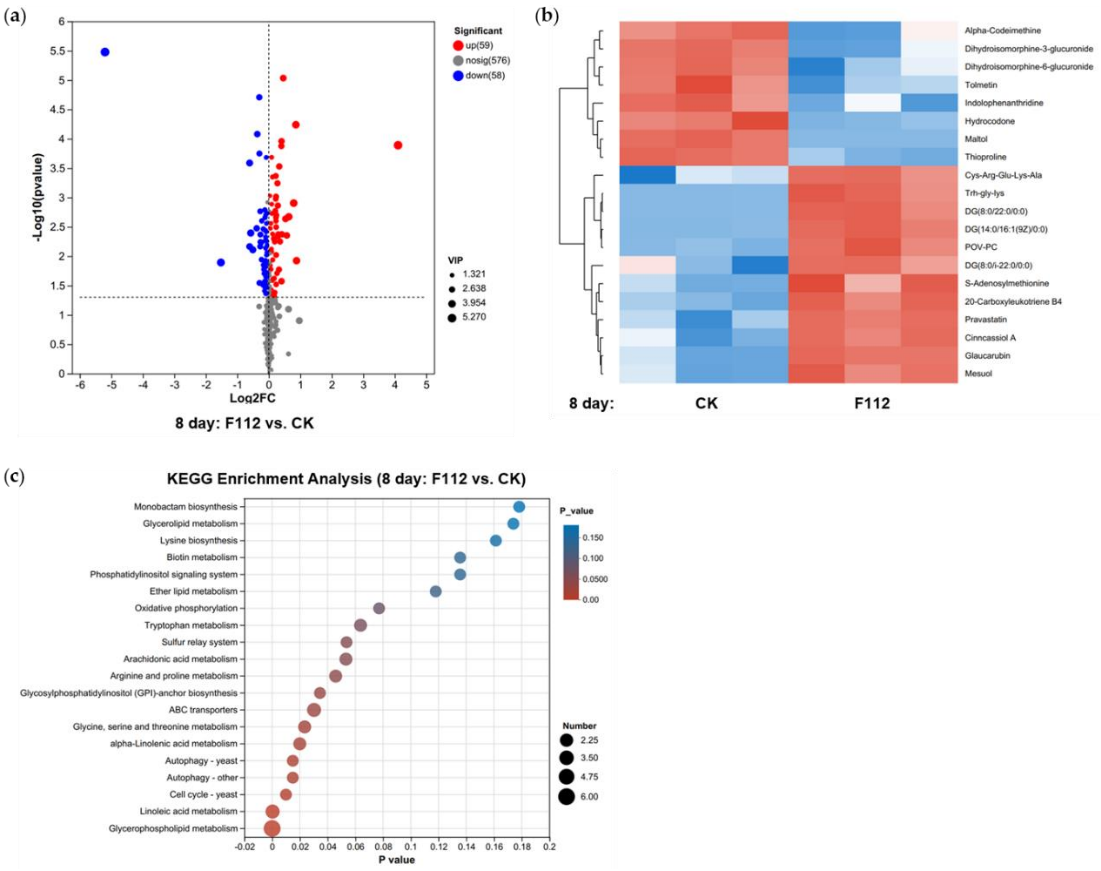

Supplementary Figure S3. Clustering Analysis of Differentially Expressed Metabolites between the F112 and CK Groups at 8 Days. (a) Volcano plots depicting differentially expressed metabolites between the F112 and CK groups at 4 days. The horizontal axis represents the log2 fold change (log2FC) in metabolite expression between the two groups, while the vertical axis represents the statistical significance of differences in metabolite expression (-log10(p_value)). Larger points indicate higher Variable Importance in Projection (VIP) values. Points on the left side represent downregulated metabolites, while points on the right side represent upregulated metabolites. The further to the left or right, and higher up on the plot, the more significant the expression difference. (b) Hierarchical clustering dendrograms illustrating the clustering of differentially expressed metabolites between the F112 and CK groups at 8 days. Closer branches indicate similar ex-pression patterns for all metabolites within the samples. Each column represents a sample, with sample names shown below. Each row represents a metabolite, with color indicating the relative expression level in the respective group, and the color gradient is depicted in the gradient color bar. (c) Pathway enrichment plots for the F112 and CK groups at 8 days. The horizontal axis represents the significance p-value of the enrichment, with lower p-values indicating greater statistical significance ($p < 0.05$ indicates significant enrichment). The vertical axis represents KEGG pathways. The size of bubbles in the plot represents the degree of enrichment of compounds in that pathway.
